# Therapeutic potential of *Ganoderma lucidum* polysaccharide peptide in Doxorubicin-induced nephropathy: modulation of renin-angiotensin system and proteinuria

**DOI:** 10.3389/fphar.2023.1287908

**Published:** 2023-09-29

**Authors:** Hui Fang, Dongmei Lin, Xinxuan Li, Lianfu Wang, Teng Yang

**Affiliations:** ^1^ Key Laboratory of Applied Pharmacology in Universities of Shandong, Department of Pharmacology, School of Pharmacy, Weifang Medical University, Weifang, Shandong, China; ^2^ National Engineering Research Center of JUNCAO Technology, Fujian Agriculture and Forestry University, Fuzhou, Fujian, China

**Keywords:** *Ganoderma lucidum* polysaccharide peptide, Doxorubicin, proteinuria, renin-angiotensin system, pro(renin) receptor

## Abstract

**Introduction:** In the Doxorubicin (DOX)-induced nephropathy model, proteinuria is a manifestation of progressive kidney injury. The pathophysiology of renal illness is heavily influenced by the renin-angiotensin system (RAS). To reduce renal RAS activation and proteinuria caused by DOX, this study evaluated the effectiveness of *Ganoderma lucidum* polysaccharide peptide (GL-PP), a new glycopeptide produced from *Ganoderma lucidum* grown on grass.

**Methods:** Three groups of BALB/c male mice were created: control, DOX, and DOX + GL-PP. GL-PP (100 mg/kg) was administered to mice by intraperitoneal injection for 4 weeks following a single intravenous injection of DOX (10 mg/kg via the tail vein).

**Results:** After 4 weeks, full-length and soluble pro(renin) receptor (fPRR/sPRR) overexpression in DOX mouse kidneys, which is crucial for the RAS pathway, was dramatically inhibited by GL-PP therapy. Additionally, GL-PP successfully reduced elevation of urinary renin activity and angiotensin II levels, supporting the idea that GL-PP inhibits RAS activation. Moreover, GL-PP showed a considerable downregulation of nicotinamide adenine nucleotide phosphate oxidase 4 (NOX4) expression and a decrease in hydrogen peroxide (H_2_O_2_) levels. GL-PP treatment effectively reduced glomerular and tubular injury induced by DOX, as evidenced by decreased proteinuria, podocyte damage, inflammation, oxidative stress, apoptosis, and fibrosis.

**Discussion:** GL-PP inhibits intrarenal PRR/sPRR-RAS activation and upregulation of NOX4 and H_2_O_2_, suggesting potential therapeutic approaches against DOX-induced nephropathy.

## 1 Introduction

Doxorubicin (DOX, Adriamycin) is a commonly used chemotherapeutic drug. However, it has deleterious effects on various organs, especially the kidneys, which limit its clinical usefulness (Liu et al., 2013). The complicated operation of the renal renin-angiotensin system (RAS) is directly related to the onset of chronic kidney disease (CKD) (Viazzi et al., 2016). Angiotensin Ⅱ (Ang Ⅱ) levels can rise as a result of DOX stimulation of renal RAS (Takenaka et al., 2017). Thus, the culmination of this cascade is the perpetuation of glomerulosclerosis, manifesting as pronounced tubulointerstitial inflammation and fibrosis. These pathological changes lead to increase urinary protein, diminished blood albumin, elevated lipid concentrations, and visible edematous swelling (Fan et al., 2015). The effectiveness of angiotensin-converting enzyme inhibitors (ACEIs) or angiotensin receptor blockers (ARBs) in reducing drug-induced hypertension and renal damage is well supported by research (Chobanian et al., 2003). Therefore, altering the activation of renal RAS may provide a workable strategy to lessen nephrotoxicity in cancer patients brought on by DOX treatment.

Proteinuria, characterized by abnormal excretion of protein in the urine, is a prominent characteristic of DOX nephropathy and a significant global healthcare concern, affecting several hundred million people worldwide (Yamashita et al., 2017). Proteinuria is frequently accompanied by increased permeability of the glomerular filtration barrier, which is primarily brought on by podocyte loss (Shi et al., 2023). This barrier acts as a highly specific filtration system within the kidneys, facilitating the elimination of waste products while retaining essential proteins in circulation. Intrarenal RAS is activated by proteinuria (Takase et al., 2005; Cao et al., 2011; Fang et al., 2018). Employing ACEIs and ARBs can mitigate proteinuria. These medications act by blocking the activation of the RAS, resulting in reduced protein leakage into urine (Chu et al., 2022). However, the efficacy of RAS blocking may be compromised by events like “Ang Ⅱ reactivation” and “aldosterone escape” that have been linked to the use of ACEIs or ARBs alone (Athyros et al., 2007). The incidence of this phenomenon is reduced when ACEIs and ARBs are administered simultaneously because this intensifies their inhibitory effects on the RAS. Nevertheless, this therapeutic combination also poses the potential unfavorable reactions such as hypotension, hyperkalemia, and renal dysfunction (Athyros et al., 2007). Consequently, exploring alternative therapeutic strategies that extend beyond RAS blockade is imperative.

The (pro) renin receptor (PRR) in the RAS is recognized as a significant regulator of blood pressure, as well as a mediator of various physiological processes (Ichihara and Yatabe, 2019). PRR has been shown to play a pivotal role in mediating the pathological effects induced by Ang Ⅱ (Wang et al., 2014), protein load (Fang et al., 2018), advanced oxidation protein products (AOPPs) (Fang et al., 2023), and DOX-induced renal disease (Luo et al., 2020). These findings highlight both the significance of PRR as a possible therapeutic target for treating renal disorders and the opportunities for innovative therapies. Site-1 protease (S1P) cleavage produces soluble PRR (sPRR), which has a variety of ramifications for both physiological and pathological processes (Fang et al., 2017; Nakagawa et al., 2017). Another important factor affecting renal function is nicotinamide adenine nucleotide phosphate (NADPH) oxidase 4 (NOX4), the predominant NADPH oxidase isoform present in renal cells (Montezano et al., 2011). Undeniably, NOX4 plays a key role in the production of reactive oxygen species (ROS), notably hydrogen peroxide (H_2_O_2_), which promotes renal oxidative stress and podocyte injury (Liang et al., 2021). The PRR inhibitor PRO20 was shown to effectively reduce proteinuria and interstitial fibrosis induced by DOX by intervening in the NOX4/ROS signaling pathway (Luo et al., 2020). Additionally, exogenous sPRR was found to stimulate NOX4-mediated ROS production, activate nuclear factor kappa-B, and induce inflammation and cell apoptosis (Fu et al., 2021). PRR, its soluble variant, sPRR, and NOX4 all act in regulating kidney inflammation, fibrosis, and oxidative stress. Intervening in these signaling pathways holds promise for potential therapy to reduce proteinuria and renal damage.

The natural medicinal fungus *Ganoderma lucidum* has been utilized in traditional Chinese medicine for over 2000 years (Meng and Yang, 2019), and demonstrates considerable pharmacological properties, including antioxidant, immunomodulatory, and anticancer activities, for the treatment and management of many illnesses such as diabetes (Ma et al., 2015), cancer (Fu et al., 2019b), and inflammation (Hu X. et al., 2020). A sizable molecular component known as *Ganoderma lucidum* polysaccharide peptide (GL-PP) has been identified and purified from the aqueous extract of *Ganoderma lucidum* fruiting bodies (Lin et al., 2003). Previous studies have demonstrated that GL-PP exhibits renoprotective properties by suppressing oxidative stress and modulating uric acid production and elimination, particularly in the setting of renal ischemia-reperfusion-induced renal failure and hyperuricemia (Zhong et al., 2015; Geng et al., 2020; Lin et al., 2022). Despite the promising results of GL-PP in the treatment of DOX-induced renal injury in mice, there remains a lack of comprehensive understanding regarding its underlying mechanisms. Here, we generated an animal model of DOX-induced kidney damage and examined the effectiveness of GL-PP in reducing proteinuria by inhibiting the RAS and NOX4/ROS signaling pathways. The results of our study provide valuable insights and novel therapeutic prospects for DOX-induced nephropathy.

## 2 Materials and methods

### 2.1 GL-PP

GL-PP, was prepared by the National Engineering Research Center of JUNCAO Technology, Fujian Agriculture and Forestry University, through extraction, separation, and reverse-phase dialysis, and was utilized *in vivo* experiments (Lin et al., 2003; Wang et al., 2007). For animal treatment, GL-PP was dissolved in normal saline.

### 2.2 Ethics statement

The experimental animals were obtained from Pengyue Experimental Animal Breeding Co., Ltd. (Jinan, China). Animal care protocols were approved by the Weifang Medical University’s Animal Care and Use Committee (License No. 2023SDL266) and followed the guidelines set by the Chinese Association for Laboratory Animal Science for the treatment and use of laboratory animals. All procedures were performed under sedation with a 2%–3% isoflurane anesthetic to minimize animal discomfort.

### 2.3 Experimental animals

Healthy male BALB/c mice, weighing 22–25 g and aged 6–7 weeks, were used in the study to examine the effect of GL-PP on renal damage. The mice were kept under strict surveillance, with regular monitoring of their health and behavior. They were housed in cages with a maximum of six animals per cage, maintaining a constant temperature of 22°C ± 2°C and a 12-h light/dark cycle. Mice had full access to distilled water and normal food. The mice used in the trials were provided by Jinan Pengyue Laboratory Animal Breeding Company (Jinan, China), and fed with rodent chow meal. The Animal Care and Use Committee of Weifang Medical University granted ethical permission for all animal studies carried out in this study, with the permit number of 2023SDL266.

### 2.4 Animal model

**FIGURE 1:**
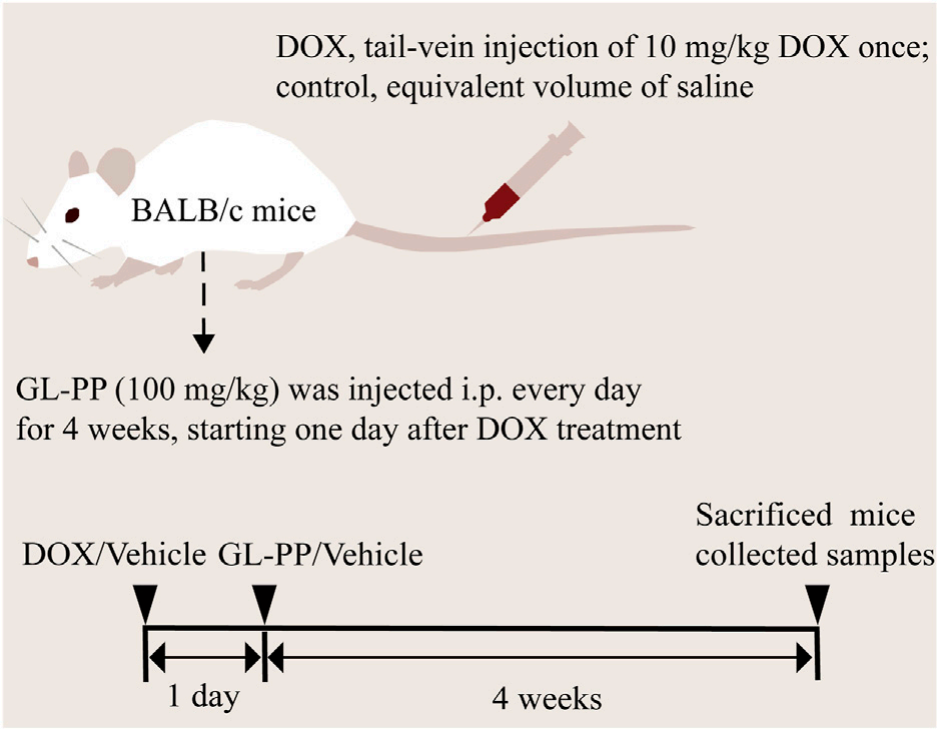
Graphical workflow of the experimental design. Abbreviations: i.p., intraperitoneal injection; GL-PP, *Ganoderma lucidum* polysaccharide peptide; DOX, Doxorubicin.

Based on the description in the previous section, the animal model was prepared (Luo et al., 2020). After acclimatizing for 1 week to controlled environmental conditions, including a temperature of 22°C ± 2°C, a 12-h light/dark cycle with standardized light intensity, humidity, and appropriate bedding, they were randomly divided into three groups of six (6) mice (Figure 1): I, the control group (CTL), which received saline (i.p.) every day for 4 weeks; Ⅱ, the DOX group, the mice were given a single-dose of 10 mg/kg DOX (tail vein injection); and Ⅲ, the DOX + GL-PP group, the mice were given a single dose of 10 mg/kg DOX (tail vein injection). GL-PP was prepared at a concentration of 16 mg/mL and 100 mg/kg GL-PP (i.p.) every day for 4 weeks. Two days after establishing the DOX model, the DOX + GL-PP group was injected with GL-PP, while the CTL group and DOX group were injected with an equal amount of saline. We collected 24-h urine samples from the mice in metabolic cages at week 0, week 2, and week 4, and recorded water intake, food consumption, and urine volume. Furthermore, we conducted weekly measurements of the body weight of the mice from week 0 to week 4 and evaluated proteinuria utilizing the Coomassie Brilliant Blue method (A045-2, Nanjing Jiancheng Bioengineering Institute, Nanjing, China), the absorbance readings were measured at 595 nm using a microplate reader (Bio-Rad, iMark, CA, USA) (Fang et al., 2018). We euthanized the mice under isoflurane anesthesia and collected kidney samples. Some of the samples were fixed in 4% paraformaldehyde, while the others were stored at −80°C. Furthermore, we used EDTA anticoagulant tubes to collect blood samples for obtaining plasma to be used for subsequent analysis.

### 2.5 Western blot

The identical steps as previously described for performing a Western Blot were followed (Fang et al., 2023). Protein determination was performed using a bicinchoninic acid protein assay kit (P0010, Beyotime, Shanghai, China) (Yi et al., 2021). The following primary antibodies were incubated overnight on the immunoblot membranes: cyclooxygenase-2 (COX-2) (AF 1924, Beyotime, Shanghai, China); inducible nitric oxide synthase (iNOS) (AF7281, Beyotime, Shanghai, China); transforming growth factor-β1 (TGF-β1) (ab31013, Abcam, Cambridge, UK); collagen I (COL-1) (GB114197, Servicebio, Wuhan, China); PRR (HPA003156, Sigma-Aldrich, Saint Louis, USA); nephrin (sc-376522, Santa Cruz, USA); podocin (sc-518088, Santa Cruz Biotechnology, California, USA); BCL2-Associated X (Bax) (GB114122, Servicebio, Wuhan, China); B-cell lymphoma-2 (Bcl-2) (GB124830, Servicebio, Wuhan, China); cysteinyl aspartate specific proteinase-3 (Caspase-3) (AF1150, Beyotime, Shanghai, China); NOX4 (AF1498, Beyotime, Shanghai, China). After washing, the membrane was incubated with secondary antibodies (SE134, Solarbio, Beijing, China) conjugated to horseradish peroxidase. Chemiluminescence (BL523A, Biosharp, HeFei, China) (Xie et al., 2022) detection was employed to visualize the protein bands, and protein expression was analyzed using the e-BLOT ECL system (Touch imager, e-blot, Shanghai, China). Quantitative analysis of the protein bands was performed using Image-Pro Plus 6.0 software, where β-actin (K200058M, Solarbio, Beijing, China) was used as an internal loading control. Detailed information about each antibody used in this experiment is taken from previously published papers (Fang et al., 2018; Lu et al., 2019; Luo et al., 2020; Wu et al., 2020; Tao et al., 2021; Zhang et al., 2021; Abdelsalam et al., 2022; Chen et al., 2022; Yu et al., 2022; Jian et al., 2023).

### 2.6 Histopathological analysis

Similar to prior descriptions (Fu et al., 2019a), pathological evaluation was conducted. Kidney tissues were fixed in 4% paraformaldehyde for 24 h, then paraffin-embedded, and cut into 5 µm slices. The kidney tissue sections were stained using the schiff periodic acid shiff (PAS) staining agent to assist in histological evaluation using a light microscope (Nexcope NIB610, Ningbo Yongxin Optics Co., Ltd., Ningbo, China). We employed a previously established scoring system for evaluating the extent of damage in the renal tubules and glomeruli (Dhande et al., 2017; Fang et al., 2023). The assessment of renal tubular injury was conducted blindly, taking into account factors such as tubular atrophy, dilation, protein casts, and infiltration of inflammatory cells. The scoring range ranged from 0 (normal) to 5 (>80% damage). Similarly, the assessment of glomerular injury was also performed blindly, with scores based on the degree of damage, ranging from 1 to 5. Grading of glomerular injury is specifically as follows: 0 for no abnormalities, 1 for mild mesangial thickening, 2 for moderate mesangial expansion without glomerular capillary thickening, 3 for severe mesangial expansion, glomerular capillary thickening, or segmental sclerosis, and 4 for more than 50% segmental sclerosis. Kidney tissue sections were observed under a bright field microscope (Nexcope NIB610, Ningbo Yongxin Optics Co., Ltd., Ningbo, China) at a magnification of 200 times using Masson’s staining, and quantitative analysis was carried out using Image J software for each kidney section. This allowed us to assess the degree of fibrosis in the kidney tissues and obtain quantitative data on the extent of fibrotic changes.

### 2.7 Biochemical parameters analysis

Plasma from mouse blood was extracted, separated, and stored at −80°C for later use. Standard reagent kits (C013-2-1, C011-2-1, A110-1-1, and A111-1-1) from Nanjing Jiancheng Bioengineering Institute (Nanjing, China) were used to measure blood urea nitrogen (BUN), blood creatinine (Bcr), and triglycerides (TG). The measurements were completed according to the guidelines and instructions provided by the manufacturer (Wang J. et al., 2021; Li et al., 2021). The absorbance readings were measured using a microplate reader (Bio-Rad, iMark, CA, USA), and BUN, Bcr and TG were measured.

### 2.8 Immunofluorescence staining

Immofluorescence staining was performed as described previously (Li D. et al., 2020). Paraffin-embedded tissue sections (described above) were processed for immunofluorescence by deparaffinizing in xylene and hydrating in a graded ethanol series. To minimize nonspecific binding, the tissue sections were blocked for 30 min with 3% bovine serum albumin (GC305010, Servicebio, Wuhan, China) dissolved with phosphate buffered saline (PBS), then incubated overnight at 4°C with primary antibodies to nephrin or podocin (Du et al., 2018). Subsequently, the slides were washed three times in PBS (G0002, Servicebio, Wuhan, China) for 5 min each time. The tissue sections were incubated with fluorescent secondary antibodies (GB25301, Servicebio, Wuhan, China) at room temperature for 50 min under light-free conditions (Hu et al., 2021). Nuclei were counterstained with 4',6-diamidino-2-phenylindole (DAPI) solution (G1012, Servicebio, Wuhan, China) at room temperature for 10 min, shielded from light (Hu J. et al., 2020). Following that, the slides were incubated in a spontaneous fluorescence quenching reagent (G1221, Servicebio, Wuhan, China) for 5 min and rinsed under water for 10 min. Finally, the film was sealed with anti-fluorescence quencher (G1401, Servicebio, Wuhan, China). Imaging was performed using a Nikon Eclipse C1 fluorescence microscope (Nikon Corporation).

### 2.9 TdT-mediated dUTP nick end labeling (TUNEL) staining

The TUNEL staining was performed as described in previous study (Wang et al., 2022). To ensure the preservation of their structural integrity, the tissues were fixed in a 4% paraformaldehyde solution for 24 h before being embedded in paraffin. The tissue slices were dewaxed to water, and then a proteinase K working solution (G1205, Servicebio, Wuhan, China) was applied and incubated at 37°C for 22 min to speed up protein breakdown (Liu et al., 2023). The tissue sections were subjected to a membrane-breaking solution (0.1% triton) and incubated at room temperature for 20 min following three PBS washes. The tissue sections were then placed in a buffer solution, washed in PBS, and incubated at room temperature for 10 min to improve the staining conditions. The tissue sections were then treated with the TUNEL reaction mixture, which includes, terminal deoxynucleotidyl transferase enzyme, 2′-deoxyuridine 5′-triphosphate, and buffer, and incubated for an hour at 37°C in a constant temperature incubator to detect apoptotic cells. To get rid of any chemicals that were not attached to the tissue sections, three cycles of PBS washing were completed. The tissue sections were stained with DAPI staining solution (G1012, Servicebio, Wuhan, China) to mark the nuclear DNA, and they were then left at room temperature in the dark for 10 min. A Nikon Eclipse C1 fluorescent microscope (Nikon Corporation) was used to capture images. Using the Image Pro Plus software, the relative fluorescence intensity of TUNEL was calculated using the formula as shown: Intensity=Total TUNEL positive signal pixel intensity / area.

### 2.10 Measurement of renin activity, Ang Ⅱ, and sPRR

Renin activity was assessed by the level of angiotensin I (Ang I) in urine samples (Fang et al., 2018). Samples were first centrifuged at 4°C and 4000 rpm for 20 min, and the resultant supernatants were collected and incubated for 1 hour at both 37°C and 4°C. According to the manufacturer’s instructions, the Ang I EIA Kit (S-1188, BMA Biomedicals, Augst, Switzerland) was used to measure the levels of Ang I (Fang et al., 2018). A commercial ELISA kit (CEA005Mu, Cloud-Clone Corp., Houston, USA) was used following the manufacturer’s instructions to measure the concentration of Ang Ⅱ in urine (Fang et al., 2018). Similar to this, the sPRR Assay Kit (27781, Immuno-Biological Laboratories, Takasaki, Japan) was used to measure the amounts of sPRR in urine without dilution (Fang et al., 2018). The absorbance readings were measured using a microplate reader (Bio-Rad, iMark, CA, USA), and renin activity, Ang Ⅱ, and sPRR were measured.

### 2.11 Measurement of thiobarbituric acid reactive substances (TBARS)

The TBARS measurement entails the interaction between thiobarbituric acid and the byproducts of lipid peroxidation, particularly malondialdehyde. The measurement of TBARS was conducted according to the guidelines outlined by the manufacturer (10009055, Cayman Chemical, Michigan, USA). The absorbance readings were measured at 530 nm using a microplate reader (Bio-Rad, iMark, CA, USA), and the amount of TBRAS was calculated (Fujita et al., 2014).

### 2.12 Measurement of H_2_O_2_

The H_2_O_2_ Assay Kit (BC3595, Solarbio, Shanghai, China) was used under the provided directions to detect H_2_O_2_. The assay is based on the development of a yellow peroxy-titanium complex as a result of the interaction between H_2_O_2_ and titanium sulfate. Microplate readers (Bio-Rad, iMark, CA, USA) were used to measure absorbance at 415 nm.

### 2.13 Measurement of superoxide dismutase (SOD), glutathione peroxidase (GSH-Px)

The manufacturer’s instructions for the specific assay kit (A001-3, A005-1-2, NanJing JianCheng Bioengineering Institute, Nanjing, China) were followed to measure the SOD and GSH-Px activities in kidney tissue samples (Xiong et al., 2020; Luo et al., 2021). These results were detected at specific wavelengths (GSH-Px: 412 nm, SOD: 450 nm), respectively, by a microplate reader (Thermo Scientific Multiskan FC; Thermo Fisher Scientific Inc., Waltham, MA).

### 2.14 Statistical analysis

An evaluation of the normality of the data was performed using the Shapiro–Wilk test. The non-parametric Kruskal-Wallis test, two-way analysis of variance (ANOVA), and one-way ANOVA were all performed using the Prism software, version 9.4 (GraphPad Software). The comparisons of data among groups were performed by Tukey’s *post hoc* test for parametric data or Dunn’s *post hoc* test for nonparametric data. Numerical results were calculated using the mean and standard error of the mean (SEM), and statistical significance was determined by *p* values <0.05 with a 95% confidence interval.

## 3 Results

### 3.1 GL-PP alleviates DOX-induced proteinuria and renal dysfunction in mice

**FIGURE 2:**
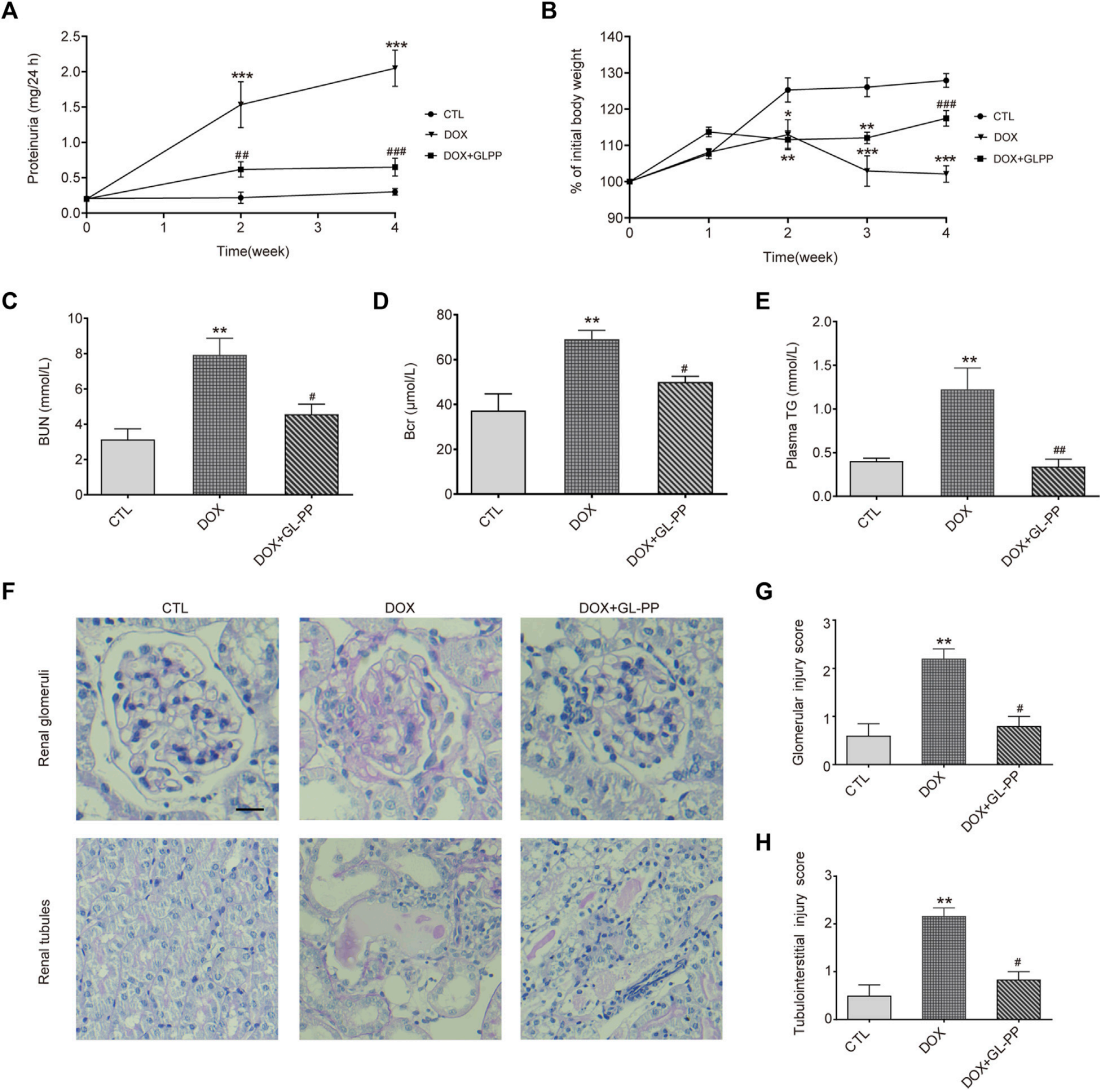
GL-PP alleviates DOX-induced proteinuria and renal dysfunction in mice. **(A)** Temporal progression of proteinuria. **(B)** Change in body weight over the course of the experiment (expressed as a percentage of initial body weight). Body weight % was computed using the formula (actual body weight/initial body weight) x 100. **(C)** Blood urea nitrogen. **(D)** Blood creatinine. **(E)** Plasma triglycerides. **(F)** Representative image of renal PAS staining (×200), scale bar = 20 μm. **(G)** Semi-quantitative scoring of glomerular injury. Calculated as the average score of 20 fields per mouse in each group. **(H)** Semi-quantitative scoring of tubulointerstitial injury, calculated as the average score of 10 fields per mouse in each group. Data are presented as mean ± SEM, N = 5-6 per group. ^*^ *p* < 0.05; ^**^ *p* < 0.01; ^***^ *p* < 0.001 vs. CTL (control); ^#^ *p* < 0.05; ^###^ *p* < 0.001 vs. DOX (Doxorubicin). For statistical analysis, **(A, B)** were analyzed using a two-way ANOVA with Tukey’s multiple comparison test. **(C, D, F)** underwent a one-way ANOVA with Tukey’s multiple comparison test. **(G ,H)** were subjected to the Kruskal-Wallis test with Dunn’s multiple comparison test.

In rodents experiencing DOX nephropathy, proteinuria and renal impairment are the hallmarks of the disease (Luo et al., 2020). Here, we examined the possible therapeutic effects of GL-PP on proteinuria using the DOX-induced nephropathy animal model. As shown in Figure 2A, following a single tail-vein injection of DOX in BALB/c mice, proteinuria gradually increased and reached its peak in the 4th week. Protein levels in urine were reduced with GL-PP treatment, starting from the 2nd week, and in the 4th week, proteinuria levels in the DOX + GL-PP group were 69% lower than of the DOX group, demonstrating that GL-PP can significantly alleviate DOX-induced proteinuria. Following a tail vein injection of DOX, body weight declined by 20% in comparison to the CTL group, reaching its lowest level in the 4th week, as depicted in Figure 2B. GL-PP facilitated the recovery of body weight by 15% in mice, showing statistical significance when compared to the DOX group. Figures 2C, D indicate that when compared to the CTL group, the DOX group’s BUN and Bcr levels increased 153% and 85%, respectively. GL-PP treatment led to a notable decrease in BUN levels by 43% and Bcr levels by 28% compared to the DOX group. Additionally, after DOX treatment, TG levels increased by 2.05×; however, GL-PP reversed DOX-induced hyperlipidemia, resulting in 72% reductions in TG levels (Figure 2E).

Furthermore, we assessed renal glomerular and tubular injury using PAS staining. Figure 2F shows that the glomeruli and tubules of the CTL group showed normal morphology, whereas the DOX group displayed thickening of the glomerular basement membrane, mesangial expansion, and adhesion of glomerular tuft, along with obvious tubular dilation, inflammatory cell infiltration, and proteinaceous casts. Scores for glomerular and tubule injuries were assigned (Figures 2G, H). The DOX group showed a significant increase of 63% and 77% in scores for glomerular and tubule injuries, respectively, compared to the CTL group. The DOX + GL-PP group, however, demonstrated a 55% and 39% reduction in glomerular and tubular tissue scores, respectively, as compared to the DOX group. Renal glomerular and tubular injury caused by DOX was ameliorated by GL-PP treatment, as evidenced by decreased tissue scores and improved morphology. Over 4 weeks, we monitored the food intake, water intake, and urine volume of all groups of mice and found no significant differences among the groups (Supplementary Table S1). Therefore, GL-PP, a potential therapeutic agent, demonstrated significant efficacy in alleviating DOX-induced proteinuria and renal dysfunction in mice.

### 3.2 GL-PP alleviates DOX-induced renal inflammation and fibrosis

**FIGURE 3:**
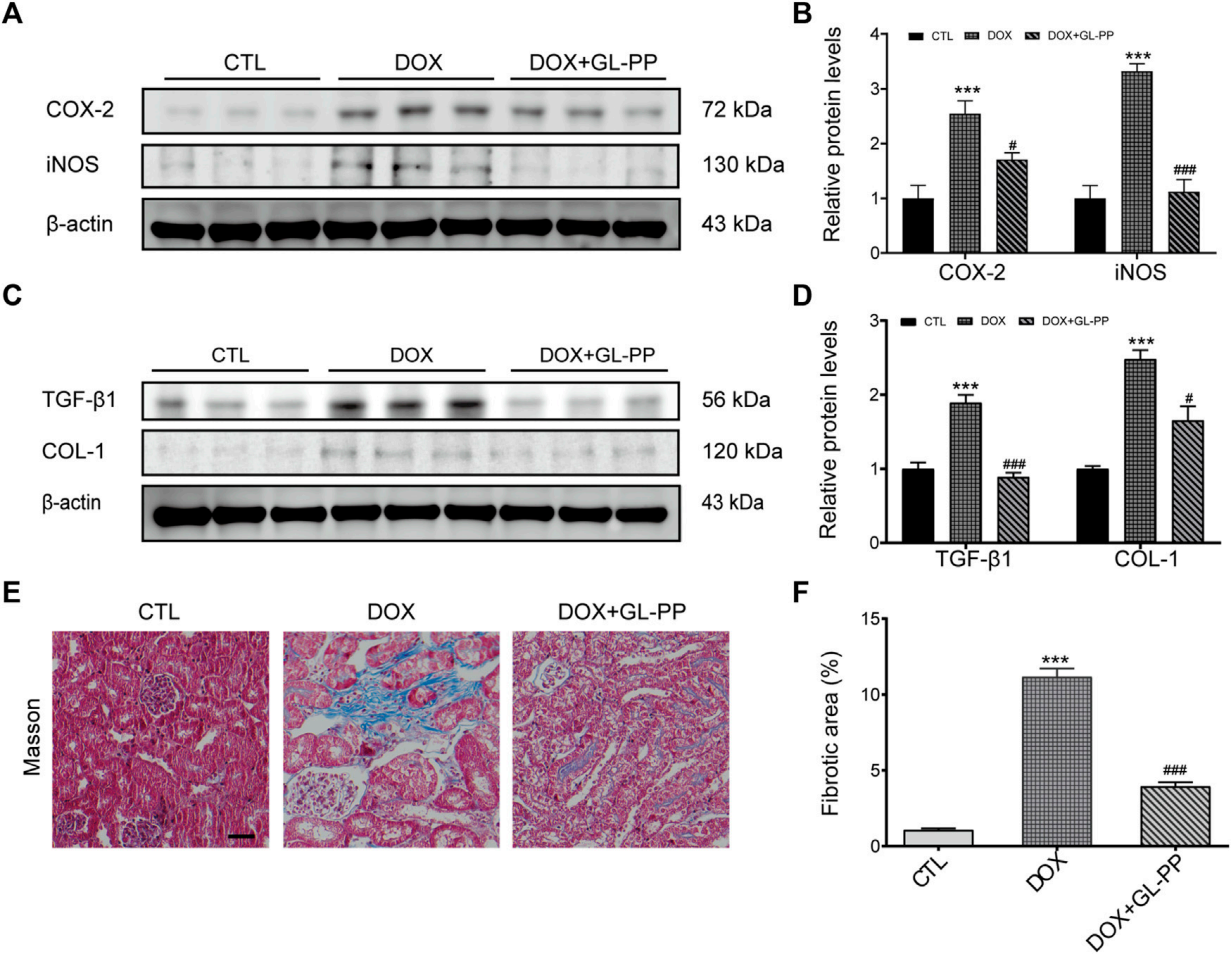
DOX-induced kidney fibrosis and inflammation are suppressed by GL-PP. **(A)** The amounts of COX-2 and iNOS proteins were assessed using Western blotting. **(B)** Quantification of the protein levels of COX-2 and iNOS adjusted to actin. **(C)** TGF-β1 and COL-1 protein levels were assessed by Western blotting. **(D)** TGF-β1 and COL-1 protein levels were measured and adjusted to β-actin. **(E)** Illustrations of Masson’s trichrome staining that are typical (×200), scale bar = 100 µm. **(F)** Masson-stained kidney slice morphometric analysis. *N* = 3-6 per group. ^**^ *p* < 0.01; ^***^ *p* < 0.001 vs. CTL (control); ^##^ *p* < 0.01; ^###^ *p* < 0.001 vs. DOX (Doxorubicin). For statistical analysis, **(B, D, F)** underwent a one-way ANOVA with Tukey’s multiple comparison test.

DOX has been shown to result in renal fibrosis and inflammation in animals (Zhou et al., 2013; Kim et al., 2019). To determine whether GL-PP could reduce inflammation and fibrosis brought on by DOX in the kidneys, we obtained the renal cortex and used Western blot analysis to measure the protein expression levels of fibrotic and pro-inflammatory molecules, including COX-2, iNOS, TGF-β1, and COL-1. According to the findings shown in Figures 3A, B, the COX-2 and iNOS protein levels were significantly higher in the DOX group as compared to the CTL group, increased 1.6 times and 2.3 times, respectively. However, the levels of the proteins COX-2 and iNOS were considerably decreased in the DOX + GL-PP group by 33% and 66%, respectively. Renal fibrosis and DOX-induced renal impairment are tightly related. When comparing the DOX group to the CTL group, we found that the expression of TGF-β1 and COL-1 proteins had significantly increased by 89% and 148%, respectively. Surprisingly, DOX-induced TGF-β1 and COL-1 protein expression were significantly reduced by treatment with GL-PP by 53% and 33%, respectively (Figures 3C, D). To further evaluate kidney fibrosis, Masson’s staining was employed. As shown in Figures 3E, F, the DOX group exhibited a significantly larger fibrotic area (blue area) compared to the CTL group, indicating DOX-induced renal fibrosis. However, GL-PP treatment resulted in a reduction of the fibrotic area within the kidney.

### 3.3 Inhibition of DOX-induced mouse renal glomerular injury by GL-PP

**FIGURE 4:**
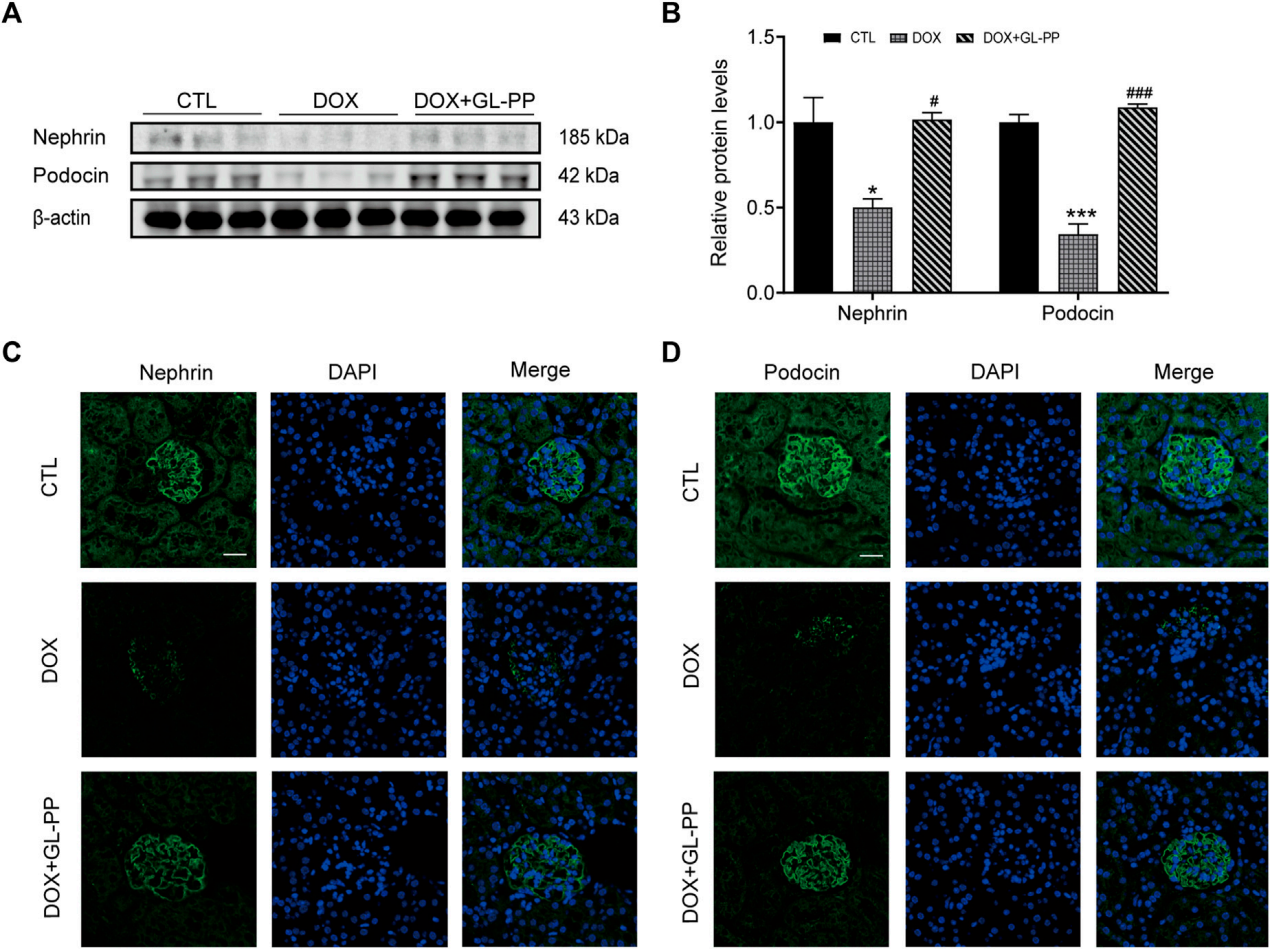
In mice, GL-PP reduces the glomerular damage brought on by DOX. **(A)** Nephrin and podocin protein levels were assessed using Western blots. **(B)** Nephrin and podocin protein levels were quantified and adjusted to β-actin in. **(C)** Nephrin immunofluorescence staining (×200), scale bar: 20 μm. **(D)** Podocin immunofluorescence staining (×200), scale bar: 20 μm. N = 3-6. ^**^ *p* < 0.01; ^***^ *p* < 0.001 vs. CTL (control); ^#^ *p* < 0.05; ^###^ *p* < 0.001 vs. DOX (Doxorubicin). For statistical analysis, Figures 4B underwent a one-way ANOVA with Tukey’s multiple comparison test.

Podocytes are an essential constituent of the renal glomerular barrier, with nephrin and podocin serving as biomarkers for evaluating podocyte injury. Both proteins significantly contribute to preventing protein translocation across the glomerular barrier (Hosoe-Nagai et al., 2017). Nephrin and podocin expression is downregulated in cases of severe podocyte injury, according to studies (Xuan et al., 2021). Similar to humans, focal segmental glomerulosclerosis (DOX nephropathy) rats show podocyte damage and decreased expression of nephrin and podocin (Li Y. et al., 2020). Nephrin and podocin protein expression were assessed to examine the effects of GL-PP. The results presented in Figures 4A, B demonstrate that compared to CTL mice, nephrin and podocin protein expression was reduced by 50% and 66% in DOX mice, respectively. However, in the DOX + GL-PP group, there was an increase of 1.0 times and 2.2 times in nephrin and podocin expression, respectively. Immunofluorescence labeling was used to further analyze the expression of nephrin and podocin in the kidney tissues of DOX mice. After being exposed to DOX, positive staining of nephrin and podocin decreased; exposure to GL-PP, however, restored their fluorescence (Figures 4C, D). These results suggest that GL-PP significantly inhibits the DOX-induced downregulation of nephrin and podocin expression.

### 3.4 Inhibition of DOX-induced mouse renal cell apoptosis by GL-PP

**FIGURE 5:**
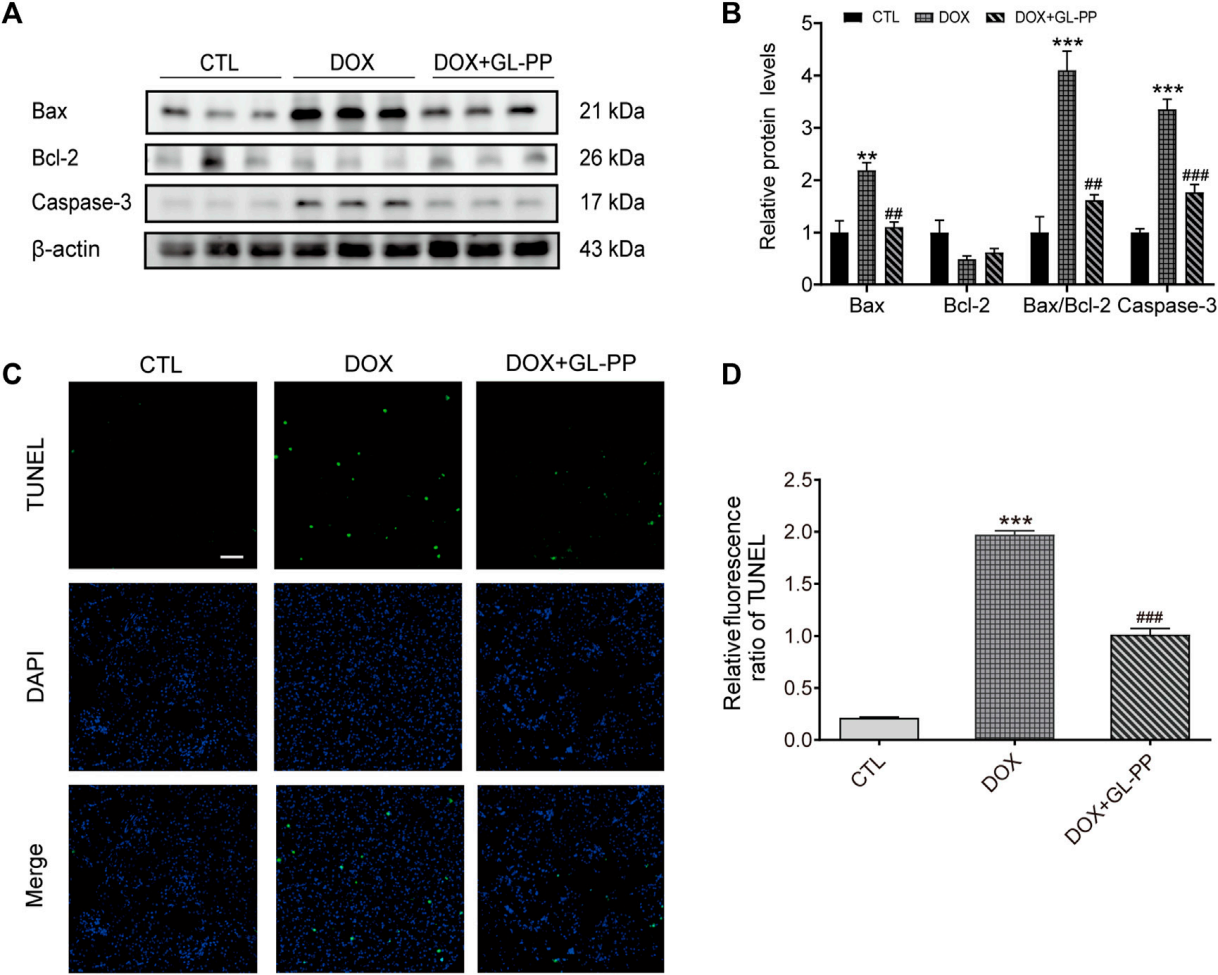
DOX-induced kidney cell death is inhibited by GL-PP. **(A)** Bax, Bcl-2, and Caspase-3 protein levels were assessed using Western blots. **(B)** Measuring the amounts of the proteins Bax, Bcl-2, Bax/Bcl-2, and Caspase-3, normalized to β-actin. **(C)** Green fluorescence TUNEL staining (×200), scale bar = 100 µm. **(D)** The TUNEL Relative Fluorescence Ratio. N = 3-6. ^**^ *p* < 0.01; ^***^ *p* < 0.001 vs. CTL (control); ^#^ *p* < 0.05; ^##^ *p* < 0.01; ^###^ *p* < 0.001 vs. DOX (Doxorubicin). For statistical analysis, **(B, D)** underwent a one-way ANOVA with Tukey’s multiple comparison test.

Previous research has shown that DOX causes apoptosis (Cai et al., 2022). To determine whether GL-PP could prevent DOX-induced cell death, we examined expression of apoptosis-related proteins after GL-PP treatment in cells exposed to DOX. GL-PP treatment reduced the expression of activated Caspase-3 protein by 47% (Figures 5A, B). Additionally, we assessed the levels of expression of the pro- and anti-apoptotic proteins Bax and Bcl-2 proteins, as the amount of cell apoptosis is determined by the ratio of Bax/Bcl-2 (Korsmeyer et al., 1993). The expression level of Bax protein was almost 2.2 × greater in the DOX group than in the CTL group Figures 5A, B. However, GL-PP therapy decreased the expression level of Bax by 50% in comparison to the DOX group. Bcl-2 protein expression did not differ significantly across any of the groups. The DOX + GL-PP group showed a 60% reduction in the Bax/Bcl-2 ratio compared to the DOX group, which is significant because the Bax/Bcl-2 ratio rose by 3.1× in the DOX group compared to the CTL group. In kidney tissues, treatment with DOX resulted in more apoptotic cells in the kidneys when compared with CTL treatment, determined by TUNEL staining. GL-PP treatment effectively reduced the TUNEL-positive staining induced by DOX, indicating its ability to protect the kidneys and alleviate DOX-induced cell apoptosis (Figures 5C, D). GL-PP apparently decreases the production of Bax and Caspase-3 to lessen the cell apoptosis brought on by DOX.

### 3.5 Inhibition of DOX-induced oxidative stress in mice by GL-PP

**FIGURE 6:**
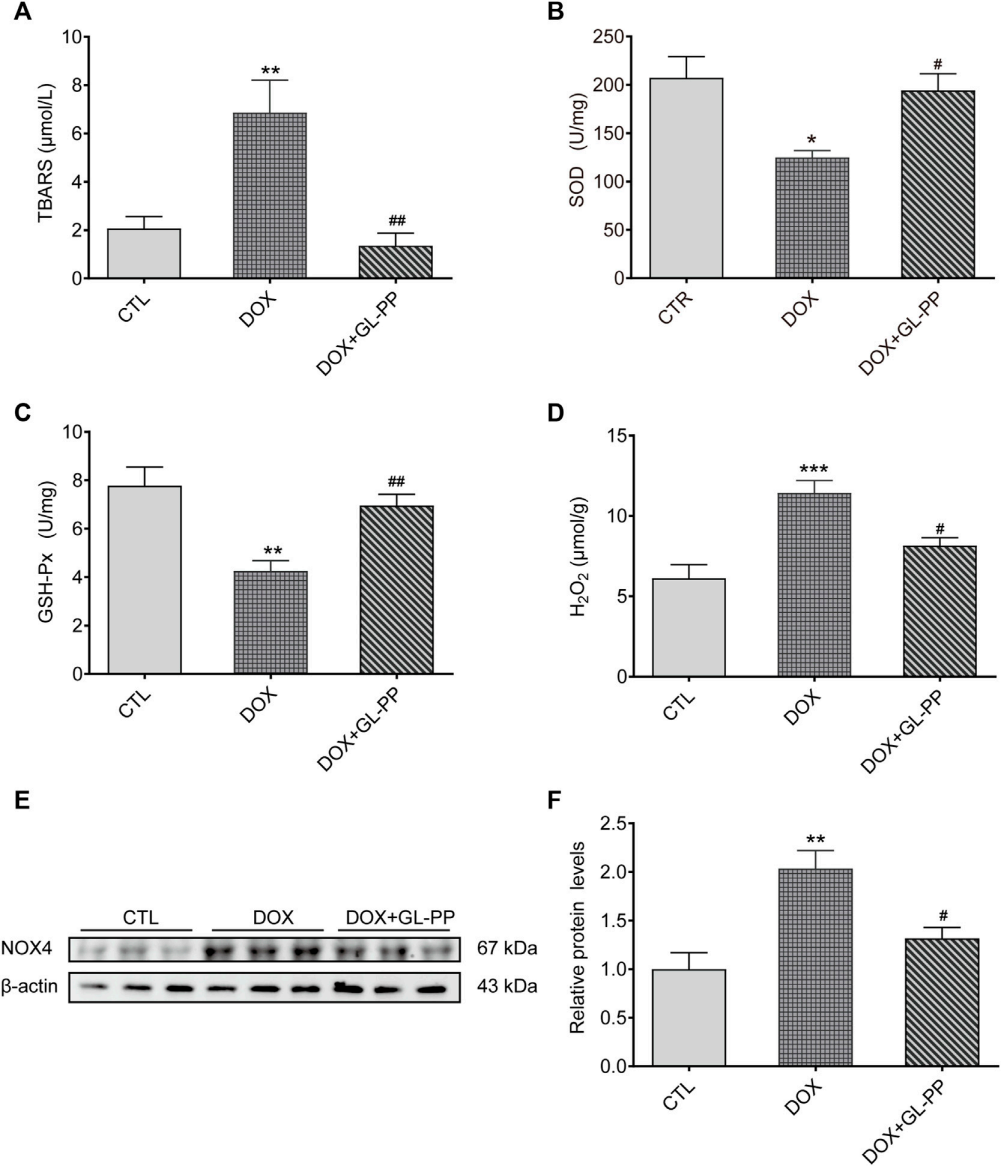
In mice, GL-PP reduces oxidative damage brought on by DOX. **(A)** Amounts of TBARS in renal tissues. **(B)** The amount of SOD in renal tissues. **(C)** GSH-Px concentrations in renal tissues. **(D)** H_2_O_2_ concentrations in renal tissues. **(E)** A Western blot was used to examine NOX4 protein levels. NOX4 protein levels were measured and adjusted to -actin in **(F)**. N = 3-6 per group. ^**^ *p* < 0.01; ^***^ *p* < 0.001 vs. CTL (control); ^#^ *p* < 0.05; ^##^ *p* < 0.01 vs. DOX (Doxorubicin). For statistical analysis, **(A, B, C, D, F)** underwent a one-way ANOVA with Tukey’s multiple comparison test.

Since oxidative stress is acknowledged to play a role in DOX-induced kidney damage (You et al., 2011), we looked at the effect of GL-PP on potential antioxidative stress in DOX-induced mice. Here, we examined TBARS, a frequently recognized indicator of oxidative stress. In contrast to the CTL group, the TBARS levels were 2× higher in the renal tissue of the DOX group (Figure 6A). DOX + GL-PP, on the other hand, resulted in a 78% reduction compared to DOX alone.

SOD and GSH-Px levels were also measured to determine the effect of GL-PP on DOX-mediated renal oxidative stress. SOD and GSH-Px levels were considerably reduced in the DOX group compared to the CTL group, but these alterations were undone by GL-PP (Figures 6B, C). In the DOX group, H_2_O_2_ content was found to be 87% higher than in the CTL group. Notably, treatment with GL-PP led to a 29% decrease in H_2_O_2_ content (Figure 6D). Additionally, NOX4 protein in the kidneys was expressed 100% higher in the DOX group compared to the CTL group. Treatment with GL-PP resulted in a 35% reduction in NOX4 protein expression in the DOX + GL-PP group (Figures 6E, F). Taken together, these findings suggest that GL-PP effectively inhibits the oxidative stress induced by DOX.

### 3.6 Inhibition of DOX-induced PRR-RAS activation in mouse kidneys by GL-PP

**FIGURE 7:**
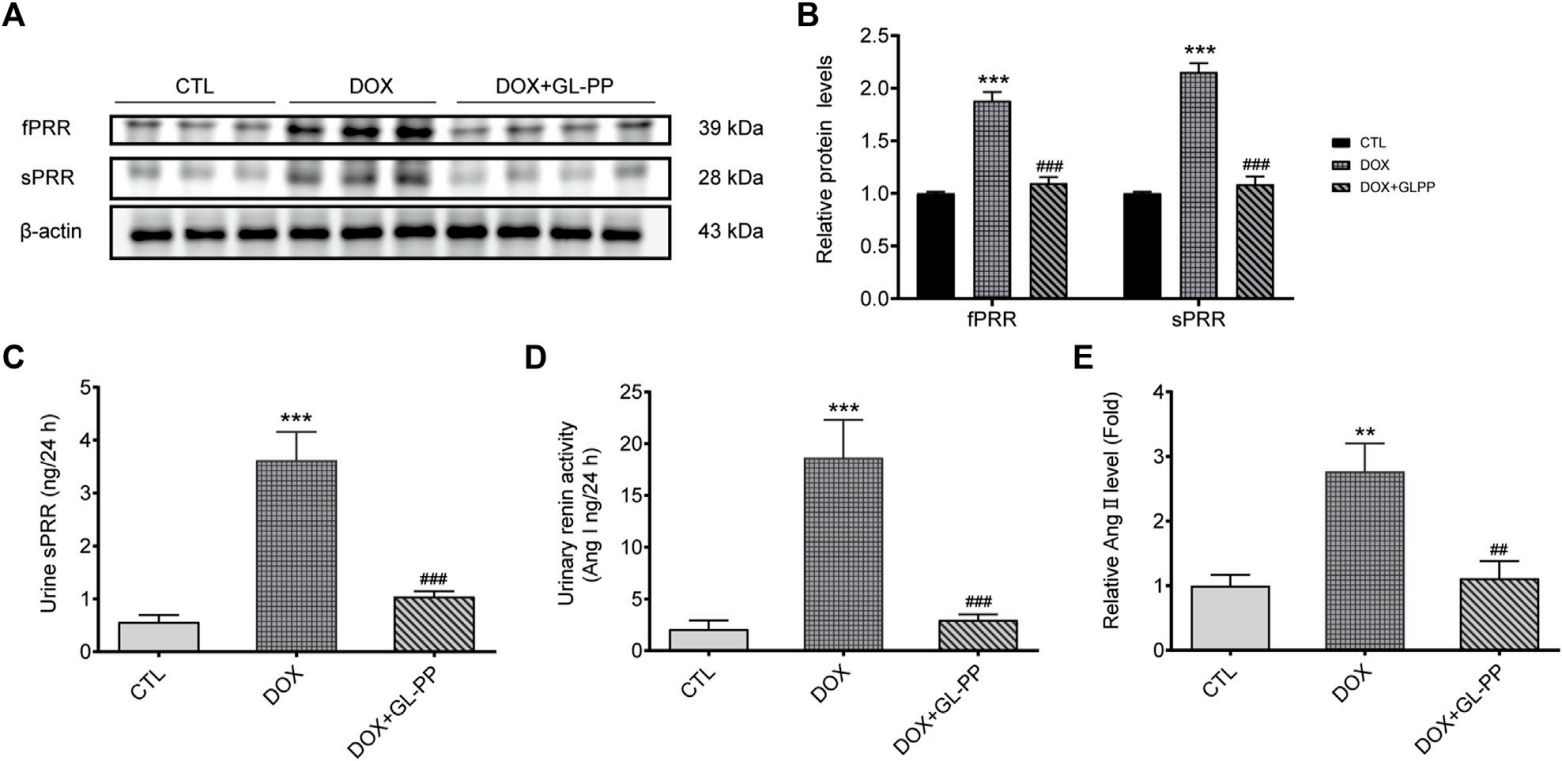
In mice with renal damage brought on by DOX, GL-PP prevents PRR-RAS activation. **(A)** The protein levels of fPRR and sPRR in the renal cortex were assessed using a Western blot. **(B)** Analysis of the protein levels of fPRR and sPRR adjusted to β-actin. **(C)** Levels of sPRR in urine. **(D)** Renin activity in the urine. **(E)** Ang Ⅱ excretion in the urine. *N* = 3–6 per group. ^***^ *p* < 0.001 vs. CTL (control); ^###^ *p* < 0.001 vs. DOX (Doxorubicin). For statistical analysis, **(B, C, D, E)** underwent a one-way ANOVA with Tukey’s multiple comparison test.

Previous reports indicated RAS is activated in mouse kidneys following DOX-induced injury (Takenaka et al., 2017). Specifically, PRR has been identified as a critical regulator of RAS activation in proteinuria kidney disease (Fang et al., 2018; Luo et al., 2020). Therefore, our objective was to investigate whether GL-PP could inhibit the upregulation of PRR/sPRR expression induced by renal RAS activation in the DOX mouse model. As shown in Figures 7A, B the expression of full-length PRR (fPRR) and sPRR rose by roughly 88% and 115% respectively, in the DOX group compared to the CTL group. Contrary to the DOX group, however, treatment with GL-PP dramatically decreased sPRR protein expression by 49% and fPRR protein expression by 41%. In addition, the concentration of sPRR in urine was quantified in both the DOX and CTL groups. Notably, a substantial elevation of sPRR in urine was observed in the DOX group, but this effect was effectively mitigated by GL-PP (Figure 7C). Additionally, we examined the impact of GL-PP on other components of the renal RAS system. As depicted in Figures 7D, E, urinary renin activity increased by 8× in the DOX group relative to the CTL group; however, it decreased by 84% in the DOX + GL-PP group. Furthermore, the urinary Ang Ⅱ levels in the DOX group were almost 2.8× higher than in the CTL group, but they reduced by roughly 60% after GL-PP therapy. These results demonstrate the effectiveness of GL-PP in the management of DOX-induced kidney illness by preventing the activation of PRR-RAS in the renal system.

## 4 Discussion

GL-PP, derived from *Ganoderma lucidum*, is a major component of traditional Asian medicine. It has a molecular weight of approximately 3.71 × 10^4^ Da and consists of polysaccharides (76.38%) and peptides (3.89%). The monosaccharide ratio includes xylose, mannose, and glucose at 4.83:1:7.03. The sugar-peptide linkage occurs through O-glycosidic bonds, mainly β-(1→3) (1→6) glucose, and also α-glycosidic bonds (Wang et al., 2015; Lin et al., 2019). GL-PP has a variety of biological actions, including anti-inflammatory, antioxidant, and anti-apoptotic properties (Geng et al., 2020). Several diseases, including hyperuricemia (Lin et al., 2022), rheumatoid arthritis (Meng et al., 2023), atherosclerosis (Wihastuti and Heriansyah, 2017), and renal, cerebral, and intestinal dysfunction (Zhang et al., 2014; Zhong et al., 2015; Lin et al., 2023), may benefit from the use of GL-PP. Based on our findings, GL-PP likely inhibits renal RAS activation, which reduces proteinuria and kidney damage brought on by DOX.

Both the beginning and progression of CKD are significantly influenced by proteinuria (Gansevoort et al., 2013), which affects both the structure and function of the glomeruli and renal tubules, reduces renal filtration function, and quickens the course of CKD (Gorriz and Martinez-Castelao, 2012). In clinical practice, commonly used drugs to treat proteinuria include ACEIs and ARBs. However, these drugs have certain limitations, such as not being suitable for all patients and having potential side effects of hyperkalemia and hypotension (McCoy et al., 2021). Evidence-based medicine supports the usefulness and safety of traditional Chinese herbal therapy in the prevention and treatment of kidney disorders (Wang X. H. et al., 2021). Comparatively, GL-PP exhibits a higher level of safety and possesses anti-inflammatory, antioxidative, and free radical clearance functions, capable of repairing and protecting kidney damage (Geng et al., 2020). Additionally, GL-PP exerts immunomodulatory effects, aiding in the promotion of physical recovery (Xie et al., 2023). Therefore, GL-PP demonstrates unique advantages in the treatment of proteinuria compared to conventional drugs.

Since DOX-induced rodent models are well-established models for studying proteinuria kidney disease (You et al., 2011), we sought to learn whether GL-PP could shield mice from the proteinuria and renal damage that VOX-induced exposure to the gas causes. After 4 weeks of DOX treatment, mice exhibited significant proteinuria and impaired renal function, including glomerular basement membrane thickening, tubular adhesion and dilation, inflammatory cell infiltration, and protein cast formation. GL-PP administration alleviated proteinuria and renal injury in DOX-treated mice. GL-PP also decreased plasma BUN, Bcr, and TG levels, reduced expression of PRR, sPRR, and NOX4 proteins, increased nephrin and podocin expression, and decreased H_2_O_2_ generation. GL-PP improved weight loss, inflammation, and oxidative stress in mice by downregulating COX-2, iNOS, COL-1, TGF-β1, Bax, and Caspase-3 expression while increasing Bcl-2, GSH-Px and SOD levels. This study highlights GL-PP’s protective effect against DOX-induced renal injury in proteinuria kidney disease by suppressing intrarenal RAS signaling and mitigating oxidative stress, apoptosis, fibrosis, and inflammation.

Heart failure, hypertension, and chronic kidney disease are just a few of the conditions that can emerge as a result of improper RAS activation (Mentz et al., 2013). Through intricate pathways including oxidative stress, Ang Ⅱ, a major RAS component, is essential in the pathophysiology of proteinuria renal disease (Kaneshiro et al., 2007). Activation of NADPH oxidase and generation of ROS in endothelial cells under Ang II stimulation may result from the upregulation of Nox2/p22phox expression and the p38 MAPK-dependent translocation of p47phox/p67phox to the cell membrane. (Liang et al., 2018). The PRR is a crucial part of the RAS, increasing renin activity by binding and activating the intrarenal RAS (Nguyen et al., 2002). PRR has been shown to exacerbate renal failure and proteinuria levels in various animal models, including the 5/6 renal nephrectomy rat model (Wang Y. et al., 2021), the protein overload rat model (Fang et al., 2018), the AOPPs-induced model (Fang et al., 2023), and the DOX-induced model (Luo et al., 2020), by activating the intrarenal RAS and causing inflammation, oxidative stress, and renal fibrosis. Additionally, higher levels of the 28 kDa product, sPRR, produced by protease-mediated cleavage of PRR, which indicates active RAS, have been seen in CKD patients (Cousin et al., 2009; Yoshikawa et al., 2011; Fang et al., 2017; Gatineau et al., 2019; Xie et al., 2020). Our recent study discovered that sPRR promotes oxidative stress response in AOPP-induced renal epithelial cells by mediating the intrarenal RAS pathway (Xue et al., 2021). Furthermore, sPRR is involved in the inflammation signal transduction induced by protein in cultured renal epithelial cells, supporting our ongoing *in vivo* research (Fang et al., 2017). In our DOX-induced nephropathy mouse model, we observed elevated expression of fPRR and sPRR proteins, increased sPRR levels, renal renin activity, and urinary Ang Ⅱ excretion. However, administration of GL-PP effectively suppressed all these mechanisms. These results imply that by blocking the PRR/sPRR-intrarenal RAS pathway, GL-PP may reduce DOX-induced oxidative stress in nephropathy.

The development of DOX nephropathy is significantly influenced by oxidative stress (You et al., 2011), and GL-PP has been shown to have an antioxidant action that protects against renal ischemia-reperfusion injury (Zhong et al., 2015). Our research shows that GL-PP reduces TBARS levels, enhances antioxidant enzyme activities, and protects the kidneys from DOX-induced damage. Notably, NOX4, which generates H_2_O_2_ and contributes to various renal diseases, is prominently expressed in the kidney (Montezano et al., 2011). Our latest investigation reveals that PRR inhibition suppresses renal RAS activation and NOX4-mediated H_2_O_2_ production, protecting the kidneys from AOPP-induced oxidative stress (Xue et al., 2021; Fang et al., 2023). In DOX mouse models, treatment with GL-PP effectively inhibits the upregulation of PRR, sPRR, and NOX4 protein expression, as well as the increase in H_2_O_2_ content, restoring the imbalance between oxidative stress and antioxidant status. In conclusion, GL-PP protects the kidneys from DOX-induced oxidative stress injury by inhibiting PRR-mediated renal RAS activation and reducing H_2_O_2_ generated by NOX4.

Inflammation and renal fibrosis are prominent features of DOX-induced renal diseases (Kim et al., 2019). Increased intrarenal RAS components are closely associated with fibrotic tissue damage (Panizo et al., 2021). Inhibiting Ang Ⅱ, a key player in tissue remodeling and fibrosis, may effectively prevent kidney inflammation (Nakamura et al., 2013). DOX induces renal fibrosis through excessive Ang Ⅱ production, TGF-β1 stimulation, and extracellular matrix accumulation (Takenaka et al., 2017). Our investigation shows that GL-PP effectively attenuates the upregulation of COX-2 and iNOS proteins, as well as TGF-β1 and COL-1 proteins in DOX-induced renal tissues. GL-PP suppresses the inflammatory response and renal fibrosis in DOX-induced kidney diseases by modulating intrarenal RAS. However, PRR silencing can inhibit NOX4 activation and the expression of fibrotic factors, suggesting that GL-PP may suppress inflammation and fibrosis through PRR-dependent NOX4 inhibition (Clavreul et al., 2011). In conclusion, GL-PP has the potential to treat DOX-induced kidney disorders by reducing inflammation and fibrosis by inhibiting PRR, regulating RAS and NOX4, and lowering the production of inflammatory fibrotic factors.

Apoptosis in cells is primarily regulated by Caspase-3, Bax, and Bcl-2 (Wu et al., 2012). In contrast to Bcl-2 inhibition of Caspase-9 and Bax promotion of apoptosis, Caspase-3 causes cell death (Nagase et al., 2002). The progression of kidney disease is accelerated by Ang Ⅱ and NOX4-induced renal cell death (Gao et al., 2021; Ning et al., 2022). Bax expression is increased and Bcl-2 expression is decreased as a result of DOX-induced kidney damage, which causes apoptosis. GL-PP reverses these effects. The Bax/Bcl-2 ratio reflects apoptotic regulation, with GL-PP reducing this ratio and suppressing Caspase-3 activation (Adams and Cory, 2007). GL-PP attenuates cellular apoptosis and offers renal protection by inhibiting RAS, reducing NOX4, and modulating the Bax/Bcl-2 ratio. In conclusion, GL-PP shows promising potential as a therapeutic intervention to mitigate renal inflammation, fibrosis, and cellular apoptosis in DOX-induced kidney diseases.

For podocyte function to be maintained, the glomerular filtration barrier must be preserved (Ye et al., 2022). Podocytes are key components that prevent the protein from passing through the glomerular barrier (Boi et al., 2023). Nephrin and podocin are important structural and signaling molecules in podocytes (Xinyun et al., 2023). Nephrin plays a role in preventing the protein from passing through the glomerular barrier by regulating multiple signaling pathways and inhibiting cell death (Zhang et al., 2019). Podocin mutations can lead to structural changes in podocytes and significant proteinuria, thus nephrin and podocin can serve as biomarkers for podocyte injury (Eid et al., 2022). Studies have shown that ARBs protect the kidneys from podocyte injury induced by renal RAS after myocardial infarction (Wen et al., 2013). Under high glucose conditions, NOX4 expression is upregulated in podocytes, leading to increased ROS production and ultimately causing podocyte injury (Yang et al., 2020). The NOX4 inhibitor GKT137831 can alleviate podocyte injury caused by diabetic nephropathy (Liang et al., 2021). Based on these findings, inhibiting RAS and NOX4 could be potential therapeutic approaches for treating podocyte injury. The results of our study show that therapy with GL-PP can reverse the downregulation of podocyte damage indicators, such as nephrin and podocin proteins, in the kidneys of DOX-induced mice, which lends more credence to this hypothesis. Immunofluorescence analysis also revealed that GL-PP restored the fluorescence attenuation of nephrin and podocin caused by DOX, indicating its potential to protect podocytes from damage.

This study has certain limitations that require further investigation. The use of a BALB/c mouse model to evaluate DOX-induced renal injury might limit the generalizability of our findings to humans due to significant biological differences. The therapeutic efficacy of GL-PP in humans must therefore be confirmed by additional clinical and preclinical research. Although our study shows that GL-PP inhibits several degenerative processes brought on by DOX, including the suppression of RAS and NOX4 expression, and a decrease in proteinuria, inflammation, oxidative stress, and fibrosis, the precise molecular pathways are yet unknown. Further research is necessary to explore the interaction between GL-PP and RAS, along with other relevant pathways. Moreover, the absence of direct comparisons between the GL-PP monotherapy group and the control group with DOX-induced pathology limits our ability to assess GL-PP’s individual effects. Nevertheless, this study provides a foundation for further exploration of GL-PP’s clinical applications. Future studies should concentrate on verifying the safety, pharmacokinetics, specific mechanisms of action, and therapeutic efficacy of GL-PP in humans.

## 5 Conclusion

**FIGURE 8:**
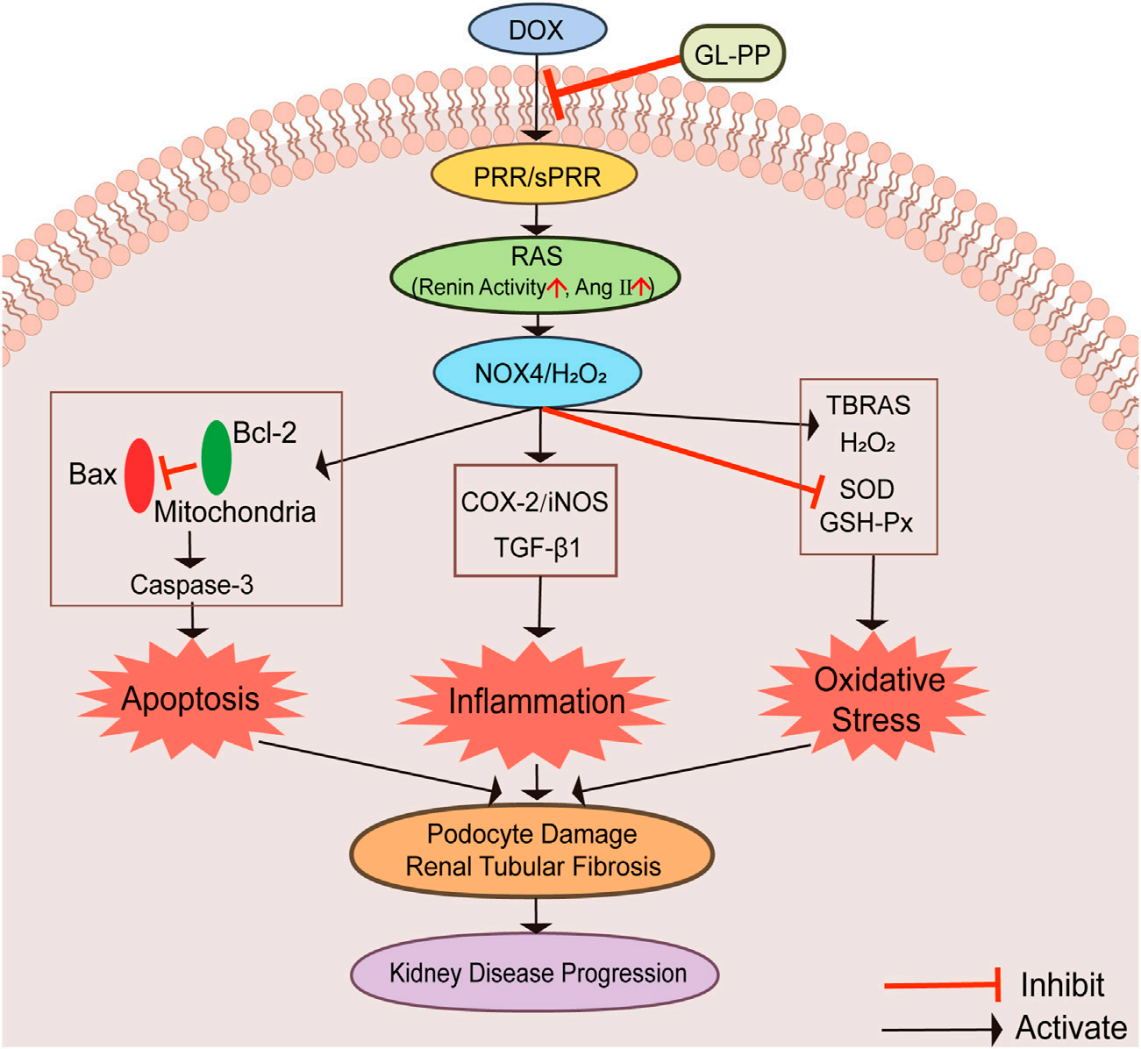
Graphical abstract illustrating the schematic diagram of the signaling pathways. Abbreviations: GL-PP, *Ganoderma lucidum* polysaccharide peptide; PRR, the (pro)renin receptor; RAS, the renin-angiotensin system; NOX4, nicotinamide adenine nucleotide phosphate oxidase 4; H_2_O_2_, hydrogen peroxide; Bax, BCL2-Associated X; Bcl-2: B-cell lymphoma-2; Caspase-3: cysteinyl aspartate specific proteinase-3; COX-2, cyclooxygenase-2; iNOS, inducible nitric oxide synthase; TGF-β1, transforming growth factor-β1; TBARS, thiobarbituric acid reactive substances; GSH-Px, glutathione peroxidase; SOD, Superoxide D.ismutase.

This study shows that GL-PP is beneficial in proteinuric kidney disease induced by DOX by suppressing the activation of PRR/sPRR-renal RAS and NOX4/H_2_O_2_ signaling pathways, which cause apoptosis, inflammation, and oxidative stress. GL-PP effectively inhibits these pathways and mitigates podocyte injury and renal tubular fibrosis, preserving renal function. Diagrams illustrating possible mechanisms are shown in Figure 8. As a promising treatment option for proteinuria kidney disease, GL-PP provides valuable options for future research and novel therapeutic strategies.

## Figures and Tables

**Table:** 

**ACEIs**	Angiotensin-converting enzyme inhibitors
**Ang I**	Angiotensin I
**Ang Ⅱ**	Angiotensin Ⅱ
**AOPPs**	Advanced oxidation protein products
**ANOVA**	Analysis of variance
**ARBs**	Angiotensin receptor blockers
**BUN**	Blood urea nitrogen
**Bcr**	Blood creatinine
**Bax**	BCL2-Associated X
**Bcl-2**	B-cell lymphoma-2
**CKD**	Chronic kidney disease
**COL-1**	Collagen I
**COX-2**	Cyclooxygenase-2
**CTL**	Control group
**Caspase-3**	Cysteinyl aspartate specific proteinase-3
**DOX**	Doxorubicin
**DAPI**	4′,6-diamidino-2-phenylindole
**fPRR**	Full-length PRR
**GL-PP**	Ganoderma lucidum polysaccharide peptide
**GSH-Px**	Glutathione peroxidase
**H2O2**	Hydrogen peroxide
**iNOS**	Inducible nitric oxide synthase
**NADPH**	Nicotinamide adenine nucleotide phosphate
**NOX4**	Nicotinamide adenine nucleotide phosphate oxidase 4
**PAS**	Schiff periodic acid shiff
**PBS**	Phosphate buffer saline
**PRR**	(pro)renin receptor
**RAS**	Renin-angiotensin system
**ROS**	Reactive oxygen species
**SEM**	Standard Error of Mean
**SOD**	Superoxide dismutase
**sPRR**	Soluble PRR
**S1P**	Site-1 protease
**TBARS**	Hiobarbituric acid reactive substances
**TGF-β1**	Transforming growth factor-β1
**TG**	Triglycerides
**TUNEL**	TdT-mediated dUTP nick end labelling

## Data Availability

The original contributions presented in the study are included in the article/Supplementary Material, further inquiries can be directed to the corresponding author.
