# Supplementary material for: Therapeutic potential of Ganoderma lucidum polysaccharide peptide in Doxorubicin-induced nephropathy: modulation of renin-angiotensin system and proteinuria
Source: Front Pharmacol. 2023 Sep 29;14:1287908. doi: 10.3389/fphar.2023.1287908 (PMC10570435; doi:10.3389/fphar.2023.1287908)
Supplement: Supplementary file 1 [file Table1.DOCX]

Supplementary Material

**Supplementary Materials**

**Table S1.** Effect of GL-PP on food intake, water intake, and urine volume in DOX mice.

| Group | Sample  Size | Week 0 | | | Week 2 | | | Week 4 | | | |
| --- | --- | --- | --- | --- | --- | --- | --- | --- | --- | --- | --- |
|  |  | Food intake  (g) | Water intake  (g) | Urine  (mL) | Food intake  (g) | Water intake  (g) | Urine  (mL) | Food intake  (g) | Water intake  (g) | Urine  (mL) |  |
| CTL | 6 | 5.3 ± 0.2 | 5.9 ± 0.3 | 0.5 ± 0.0 | 4.7 ± 0.4 | 5.8 ± 0.7 | 0.5 ± 0.1 | 3.7 ± 0.3 | 6.0 ± 1.0 | 0.6 ± 0.1 |  |
| DOX | 6 | 4.3 ± 0.3 | 6.0 ± 1.3 | 0.6 ± 0.1 | 4.1 ± 0.2 | 4.4 ± 0.5 | 0.7 ± 0.1 | 2.8 ± 0.6 | 3.9 ± 0.4 | 0.7 ± 0.1 |  |
| DOX + GL-PP | 6 | 4.2 ± 0.4 | 4.8 ± 0.8 | 0.6 ± 0.1 | 3.9 ± 0.7 | 4.6 ± 0.9 | 0.4 ± 0.1 | 3.3 ± 0.3 | 3.4 ± 0.5 | 0.3 ± 0.1 |  |
